# Cochlear implantation as a treatment for single-sided deafness and asymmetric hearing loss: a randomized controlled evaluation of cost-utility

**DOI:** 10.1186/s12901-019-0066-7

**Published:** 2019-02-04

**Authors:** Mathieu Marx, Nadège Costa, Benoit Lepage, Soumia Taoui, Laurent Molinier, Olivier Deguine, Bernard Fraysse

**Affiliations:** 1 Service d’Oto–Rhino–Laryngologie, d’Oto-Neurologie et d’ORL Pédiatrique, Centre Hospitalier Universitaire de Toulouse, Place du Dr Baylac, 31059 Toulouse Cedex 9, France; 2 Université de Toulouse, CerCo UMR 5549 CNRS, Université Paul Sabatier, Place du Dr Baylac, 31059 Toulouse Cedex 9, France; 3 Health Economic Unit, Centre Hospitalier Universitaire de Toulouse, Hôtel-Dieu Saint-Jacques, 2, rue viguerie, 31059 Toulouse Cedex 9, France; 4 Unité Inserm UMR 1027, Faculté de Médecine, National Institute for Health and Medical Research (Inserm), 37 allées Jules Guesde, 31073 Toulouse, France; 5 Department of Epidemiology, USMR, 37 allées Jules Guesde, 31073 Toulouse, France

**Keywords:** Cochlear implant, Single-sided deafness, Asymmetric hearing loss, Tinnitus, Binaural hearing, Cost-utility

## Abstract

**Background:**

Single-sided deafness (SSD) and asymmetric hearing loss (AHL) have recently been proposed as a new indication for cochlear implantation. There is still no recommended treatment for these hearing deficits, and most options considered rely on the transfer of sound from the poor ear to the better ear, using Contralateral Routing of the Signal (CROS) hearing aids or bone conduction (BC) devices. In contrast, cochlear implantation allows the poor ear to be stimulated and binaural hearing abilities to be partially restored. Indeed, most recently published studies have reported an improvement in the spatial localisation of an incoming sound and better speech recognition in noisy environments after cochlear implantation in SSD/AHL subjects. It also provides consistent relief of tinnitus when associated. These encouraging hearing outcomes raise the question of the cost-utility of this expensive treatment in an extended indication.

**Methods:**

The final endpoint of this national multicentre study is to determine the incremental cost-utility ratio (ICUR) of cochlear implantation in comparison to the current standard of care in France through simple observation, using a randomised controlled trial. Firstly, the study comprises a prospective and descriptive part, where 150 SSD/AHL subjects try CROS hearing aids and a BC device for three weeks each. Secondly, the choice is made between CROS hearing aids, BC implanted device and cochlear implantation. Hearing outcomes and quality of life measurements are described after 6 months for the subjects who chose CROS, BC or declined any option. The subjects who opt for cochlear implantation are randomised between one group where the cochlear implant is inserted without delay and one group of simple initial observation. Hearing outcomes and quality of life measurements are compared after 6 months.

**Discussion:**

The present study was designed to assess the efficiency of cochlear implantation in SSD/AHL. A favourable cost-utility ratio in this extended indication would strengthen the promising clinical results and justify a reimbursement by the health insurance. The efficiency of other options (CROS, BC) will also be described.

**Trial registration:**

This research has been registered in ClinicalTrials.gov (http://www.clinicaltrials.gov/), the 29th July 2014 under the n°NCT02204618.

## Background

Treatment of single-sided deafness (SSD) and asymmetric hearing loss (AHL) is a recurrent focus of interest in contemporary otorhinolaryngology because their consequences were globally underestimated until the late 1970’s, by both the general public and professionals of auditory disorders, assuming that the good ear would compensate the deficit of the poor ear. Although the level of handicap generated varies, SSD and AHL are actually disabling conditions in both children and adults. In the paediatric population, several case-control studies have demonstrated the impact of such hearing deficits on cognitive functions [1, 2] and schooling with higher rates of grade failure [3–5]. In adults, quality of life is often assessed using the Speech, Spatial and Qualities Hearing scale (SSQ) [6] and it has been repeatedly shown that more than 75% of SSD/AHL subjects experience hearing difficulties [7, 8]. Interaural asymmetry of hearing is the common factor to SSD and AHL, with SSD being defined as a unilateral severe-to-profound deafness (pure-tone average PTA ≥70 dB HL), with a better, normal or near-normal ear (PTA ≤ 30 dB HL). The term “AHL” is used when hearing in the better ear is not normal, but can be restored using a conventional hearing aid (PTA between 30 dB HL and 55–60 dB HL) [9, 10].

From a physiological point of view, the deficit produced by SSD and AHL is first related to the disruption of binaural hearing and its binaural advantages. Indeed, in normal hearing subjects, an incoming sound arrives with differences between the two ears in terms of timing (interaural time difference) and amplitude (interaural level difference), depending on its spatial position and spectral content (see [11] for review). These interaural differences can be used to localise a sound source in the horizontal plane and generate binaural effects. Three main effects are described, one being purely peripheral while the two others arise from the central auditory system. Thus, the head shadow effect is mainly related to the physical presence of the head, which reduces the competing noise for the ear closer to the sound source. Summation and binaural unmasking are the two central effects generated to optimise the global signal/noise ratio (SNR) if speech and noise are collocated (summation) or spatially separated (binaural unmasking or squelch). Altogether, these processes facilitate speech recognition in noisy environments which is one of the main complaints of SSD/AHL patients [12, 13].

But the loss of binaural hearing is often not isolated and several disabling symptoms can accompany the unilateral profound deafness. Tinnitus is probably the most frequently reported, affecting 67 to 100% of subjects with profound deafness [14, 15]. Because it can lead to severe psychological comorbidities such as depression and anxiety, it may be the most prominent complaint in some SSD/AHL patients [16–18]. Hyperacusia can also be associated with the symptoms and further impairs social interaction in noisy places [19].

The treatment of SSD and AHL is still debated. Although Controlateral Routing of the Signal (CROS) hearing aids system are considered to be the standard care in several countries, French authorities have not yet recommended any official guideline. Clinical experience shows that some patients adapt to this condition or simply use a conventional hearing aid for the better ear in cases of AHL [8], but recent consensus papers have suggested that trials of more specific treatments should be included in the assessment protocol [9, 10]. Among the different specific treatment options, one strategy indeed relies on the transfer of the sound surrounding the poor ear to the better ear so that it can be perceived, through medical devices such as CROS hearing aids or Bone Conduction (BC) devices. CROS hearing aids thus transmit the sound via Bluetooth. The most widespread BC devices are bone-anchored hearing aids (BAHA), which use the conductive properties of the skull to convey sounds to the better ear. Both devices allow better recognition when speech is presented to the poor ear, even in noisy settings [20–22]. In some subjects, this can lead to significant improvements in the appreciation of the quality of sounds perceived in background noise [23]. But these devices only provide a unilateral auditory input by stimulating the better ear and, consequently, cannot restore binaural hearing. In the same way, the absence of auditory stimulation in the poor ear explains why the impact on tinnitus, when associated, is merely assessed and most plausibly absent [9].

Although cochlear implantation is traditionally used to treat bilateral profound deafness, it has recently been proposed as a new treatment option in SSD/AHL, specifically because it ensures the stimulation of the poor ear. Bilateral auditory input can therefore be restored in those patients, which is a major difference to CROS hearing aids and BC devices and is the theoretical prerequisite for an improvement of binaural hearing. Furthermore, the beneficial effect of cochlear implant on tinnitus has been consistently demonstrated in numerous studies on bilateral profound deafness [24] and can be expected in SSD/AHL subjects. The treatment of tinnitus was in fact the main objective of the first report on cochlear implantation in this population [18] and the initial promising results were confirmed by other teams [25, 26] and maintained several years after the procedure with a significant relief observed in the vast majority of subjects. Early studies on binaural hearing abilities after cochlear implantation showed, on average, an improvement for localisation performances [27] and speech recognition in noise with the CI “on” compared to the CI “off” condition, with a significant inter-individual heterogeneity. Recent reviews of the literature and meta-analyses have underlined the effect of CI use on tinnitus relief, which appears more significant and consistent than the modifications of binaural hearing [28, 29]. An emphasis has also been put on the need for larger prospective randomised controlled studies to improve the level of evidence for CI efficiency in SSD/AHL.

In a prospective pilot study, Arndt et al. [25] showed that CI provided better performance than CROS or BAHAs previously tested by the same subjects, for several assessments of binaural hearing abilities. Several national multicentre trials have begun to compare hearing results after CI to those obtained with a CROS or BAHA system [30, 31]. These trials also assess the utility of the different treatments as a secondary objective, through their impact on quality of life with respect to their cost. The question of the cost/utility ratio of each treatment should indeed be raised because it is estimated that the global cost of cochlear implantation is approximately EUR 30,000 [32] while rehabilitation by CROS hearing aids or BAHA may cost EUR 2000 to EUR 3800. This gap between devices in terms of cost adds another substantial difference between CROS systems, BAHA and a cochlear implantation. Furthermore, treatment with cochlear implant involves a significant commitment on the patients’ part for the surgical procedure as well as for the rehabilitation programme; and although treatment by CROS hearing aids is not trivial, its consequences are much less significant.

All these differences between the possible treatments of SSD/AHL probably make cochlear implantation an option that is set apart from the others. Therefore in the recent consensus papers, it is not considered as an alternative among different options, but as a second-line treatment, in the event CROS or BAHA systems fail.

**Fig. 1:**
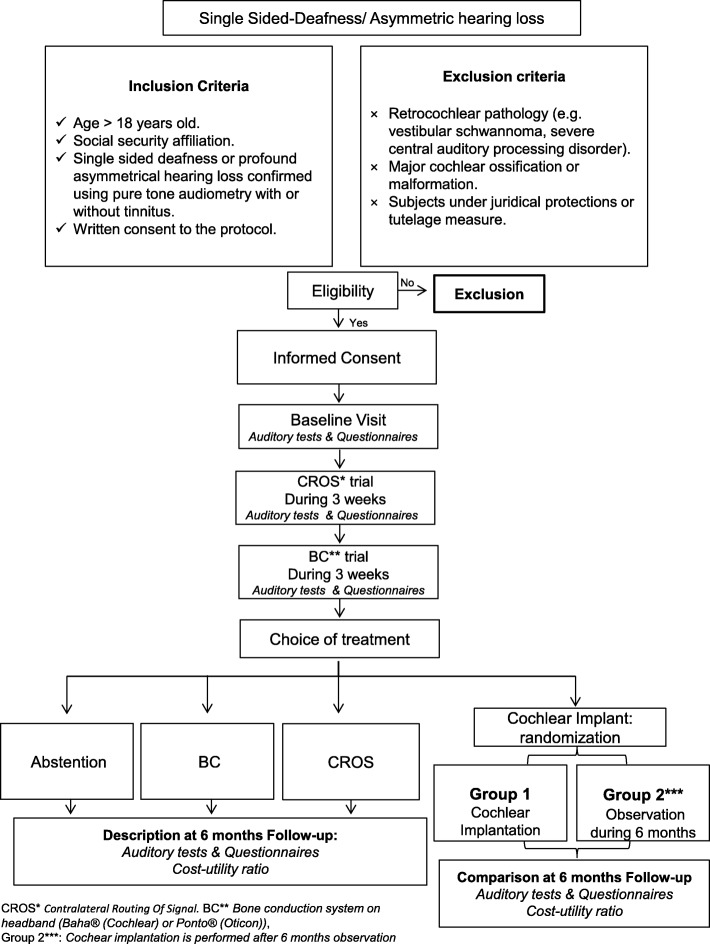
Trial design:the choice of the treatment option is made after a consecutive trial of CROS hearing aids and BAHA on a headband; subjects who opt for a cochlear implant are randomized between initial observation and cochlear implantation

The present article describes a prospective multicentre randomised controlled trial initiated in France, which allows the subjects to choose their treatment option (observation, CROS, BAHA, or CI). The decision was made to randomise CI versus observation after a consecutive trial of CROS hearing aids and BAHA on a headband (Baha® or Ponto®) (see Fig. 1). Eventually, it aims to determine if CI is cost-effective in SSD/AHL, in subjects where CROS hearing aids and BAHA trials have failed.

## Study objectives

### Primary objective

The main objective of this study is to assess the efficiency of CI in SSD/AHL, compared to the current standard of care in France (observation), after failure of CROS and BC trials, using a cost-utility analysis.

### Secondary objectives

This study also aims to:

- assess the changes in quality of life and binaural hearing performance for each successive intervention (CROS, BC and CI);
- search for associations between changes in hearing performance scores and changes in quality of life for each intervention (CROS, BC and CI);
- assess the evolution of tinnitus intensity and the level of annoyance for each intervention (CROS, BC and CI).

The cost-utility ratio of other treatment options (CROS, BC) will be determined. Hearing outcomes of each option (observation, CROS, BC, CI) will be described using tests for speech recognition in noise and for horizontal localisation.

The affordability to the French Health Insurance of the widespread implementation of CI in SSD/AHL will be assessed.

## Methods

### Study design and setting

This prospective multicentre national study combines two major steps:

- a prospective, descriptive observational cohort study, with a 6-month follow-up for SSD/AHL adult subjects treated by CROS, SSD/AHL subjects treated by an implanted bone conduction device (Baha or Ponto), and adult patients who decline all the options.

- and an open-label randomised controlled clinical trial for adults with SSD or AHL after failure of both CROS and BC systems, in two parallel groups: Observation for 6 months versus Cochlear implantation.

Participants are recruited in 7 tertiary referral centres in France. Once the inclusion/criteria are checked and the consent from the subject is obtained by one of the investigators, a reference evaluation is made for quality of life, binaural hearing performances (i.e. speech recognition in noise and horizontal localisation) and tinnitus severity. Then, CROS hearing aids and a BC device on a headband are successively tried by the subjects for three weeks each. The evaluation of quality of life, binaural hearing abilities, and tinnitus severity is repeated after the first trial (CROS) and the second trial (BC). At this point, the subject may choose the treatment he/she is willing to continue. If one of the trials (CROS or BC) is considered to be successful and the subject is willing to continue with the corresponding device, it is then provided and a new evaluation is performed 6 months later. If none of the trials is considered to be successful and the subject does not want any other treatment, then only a new evaluation is performed 6 months later. If none of the trials is considered to be successful and the subject wants treatment by cochlear implantation, the second major step of the study begins with the randomisation procedure.

Subjects who chose a cochlear implant are thus randomised between two arms: in the first one (“CI” arm), a cochlear implantation is performed within a month and a new evaluation on quality of life, binaural hearing performances, and tinnitus severity is planned 6 months after CI activation. Subjects of the second arm (“observation” arm) simply undergo the new evaluation 6 months after randomisation, without any intercurrent treatment for SSD/AHL. For obvious ethical reasons, subjects of the second arm benefit from a cochlear implantation after this 6-month evaluation.

Data from all the subjects included will be anonymised, collected and analysed. If a serious adverse event does not allow the end of the protocol to be reached and/or if a subject withdraws his/her consent, the data which were collected until this event will also be taken into account in the final analysis. If a serious adverse event occurs, it will be reported to the coordinating centre (Toulouse University Hospital) and to the “Agence Nationale de Sécurité du Médicament” (ANSM, National Agency of Drug Safety).

### Inclusion criteria

Age eighteen years or older.

French speaking.

French social security affiliation.

Ability and willingness to participate in all the assessments, with written consent to the protocol.

Poorer ear: severe-to-profound hearing loss measured using pure tone audiometry with pure-tone average (0.5, 1, 2, 4 kHz) ≥ 70 dB, confirmed by auditory brainstem responses, with minimal benefit of a conventional hearing aid. In order to account for the heterogeneity of this population, no selection was applied on the duration of deafness or the severity of the tinnitus.

Better ear: normal hearing thresholds (≤20 dB HL) to moderate hearing loss (PTA ≤ 60 dB HL).

### Exclusion criteria

Retrocochlear pathology (vestibular schwannoma, severe central auditory processing disorder).

Major cochlear ossification or malformation which could prevent the full insertion of a cochlear implant, controlled on CT scan and MRI of temporal bones.

Subjects under juridical protections or tutelage measure.

Neurological or psychiatric disorder.

Previous treatment with CROS, BC or CI.

Medical condition that contraindicates CI surgery.

### Randomisation procedure

Randomisation of CI versus observation was based on a 1:1 ratio and was stratified per centre. The allocation sequence was randomly generated by a computer (Stata SE 11.2, ralloc procedure), providing a sequence of treatments randomly permuted in blocks of varying size [2, 4, 6]. There was no blinding procedure in this open-label trial; however randomisation was centralised at the Toulouse University Hospital and managed by the project manager and clinical research associates. The allocation sequence was unknown to the investigators who enrolled participants.

### Population and sample size

The sample size was calculated based on the expected number of failures of CROS hearing aids, BC trials and on the expected improvement of quality of life in cochlear implanted subjects with SSD/AHL. In a pilot study performed in our centre (unpublished), 40 to 50% of SSD/AHL subjects declined both CROS and BC devices after a three-week trial. This rate is consistent with previous studies performed on BC implantation after a headband trial (45% in Desmet et al. [33]). An improvement in quality of life is expected in these subjects after cochlear implantation, but it is not as significant as in patients with bilateral severe-to-profound deafness. A recent meta-analysis performed on traditional CI candidates with severe-to-profound deafness demonstrated an improvement of 1.05 standard deviations. To detect an improvement of 0.8 standard deviations with an alpha risk of 5% and an 80% power, 50 subjects (25 per arm) are necessary. Altogether, and to compensate the risk of dropouts, 150 subjects are included in this study.

### Interventions

The purpose of this study is to assess the cost-utility ratio of CI in subjects with SSD/AHL after failure of more conventional treatments. Therefore, every subject will successively try CROS hearing aids and a BC device placed on a headband, for three weeks each. The baseline assessment of each trial relies on auditory measures (speech recognition in noise and horizontal localisation) and quality of life questionnaires. Once each trial has been performed, the subject will decide which treatment he/she opts for. This choice is mainly based on the subjective feelings of the subject after the two trials, but also guided by the auditory outcomes obtained with each device, and the counselling of the physician. The programme provides all costs directly related to the treatment, i.e. the cost of the medical device and the following fitting sessions.

The information regarding the results which may be expected from a cochlear implant is critical. A CI is the only device which cannot be herein tried before the decision is made and its choice intrinsically relies on hedging a bet. Across the seven centres, this information is standardised through several elements indicated to the subjects willing to try a CI, based on the literature on cochlear implantation outcomes in SSD/AHL [28, 29, 34, 35]:

- CI may restore binaural hearing to some extent but with a significant inter-individual variability;
- its effect is more consistent on ipsilateral tinnitus when associated with unilateral profound deafness;
- The impact of CI in this indication has mainly been studied and demonstrated in subjects where the duration of profound deafness was less than 10 years.

Once this information has been delivered, each subject makes a final choice among four options: abstention, CROS hearing aids, Bone-Anchored hearing aid (Baha or Ponto), or CI. Subjects who chose CI are randomised between two arms (“CI” arm versus “observation” arm) and re-assessed and compared after 6 months their choice has been implemented, and the results of the two arms are compared. Subjects who chose abstention, CROS or BAHA are re-assessed 6 months after their choice has been implemented, and the results of each group are described.

### Abstention

Subjects in this group who do not experience any benefit from the CROS hearing aid trial or from the BAHA trial, and choose no specific treatment for SSD/AHL.

### CROS hearing aids

In CROS systems, a remote microphone which is placed on the poorer ear receives the surrounding incoming signal and transmits it to the second hearing aid on the better ear, using a wireless link. The second hearing aid delivers the signal using air conduction and can also apply an additional amplification in case of hearing loss in the better ear.

A Phonak CROS system will be used for the trials and in case the subject eventually opts for this type of rehabilitation (Phonak AG, Stäfa, Switzerland). The hearing aids will be fitted by an experienced audiologist, using the fitting software provided by the company (Phonak Target™ 3.0).

### Bone conduction device (BAHA)

Bone-anchored hearing aids transmit the auditory signal received by the processor placed behind the poor ear to the better ear through bone conduction of the skull. The programme does not provide any additional hearing aid in the better ear if the subject has AHL.

Baha BP 110® (Cochlear Ltd) and Ponto® (Oticon) will be used on a headband for the trial period and placed on the corresponding abutment after a surgical procedure if the subject chooses this option. The surgery consists of the placement of the implant which aims at being osteo-integrated, on which an abutment is screwed. The processor may be used for 3 to 6 weeks following the surgery according to wound healing. It is then fitted by an experienced audiologist following the company’s guidelines.

### Cochlear implant

A surgical procedure is performed to allow the placement of the receptor under the temporal muscle and the introduction of the electrode array into the scala tympani via the round window or a cochleostomy. According to the company, the electrode array supports 12 to 22 intra-cochlear electrodes which aim at stimulating the first neurons of the auditory nerve. Four cochlear implant companies are involved in this study: Advanced Bionics, Cochlear, MedEl and Oticon. The choice of the brand will depend on the physician in charge of the patient. Approximately four weeks after surgery, the external processor can be activated and the patient will therefore receive electric auditory stimulation of the implanted ear.

### Primary outcome measure

The primary outcome measure will be applied to the two randomised arms to determine the incremental cost-utility ratio (ICUR) of cochlear implantation versus initial observation at 6 months. The utility is defined in this study as survival weighted by the quality of life of patients at 6 months (QALY). Quality of life will be measured using a generic questionnaire, the EuroQol-5D-3 L scale. This instrument is recommended by the national French agency (Haute Autorité de Santé) for cost-utility studies [36].

The EQ-5D-3 L is a self-administered, generic and multidimensional questionnaire [37]. It has two components, a descriptive component and a visual analogue scale (EQ-VAS). The descriptive component is composed of 5 dimensions described by three levels that define 243 health states. The EQ-5D-3 L is translated and validated into the French language and it has a utility function calculated on the basis of known preferences of the French population [38]. The utility-preference approach of the EQ-5D-3 L provides a cardinal measuring instrument available to calculate the cost-utility ratios.

Healthcare costs will be assessed from the French health insurance perspective [39, 40]. Direct medical and non-medical costs will be included in this study. Costs related to productivity loss will also be analysed. Direct medical costs correspond to hospitalisation costs, outpatient costs (i.e. visits and medical acts, paramedical acts), medication and medical device costs, especially the cost of the CI. Non-medical costs include transportation costs. Data linked to the number of days the patient has missed work will also be gathered from the French health insurance databases. Costs will be estimated by multiplying the number of units used for each resource with the corresponding unit cost.

Consumption of healthcare resources will be retrospectively gathered from the French Social Health Insurance databases, using a bottom-up approach. Administrative data corresponding to the name, surname, date of birth, place of living and gender were recorded for patients included in the study.

Inpatient stays will be valued using the French Disease Related Groups (DRGs). Outpatient care, which includes visits, medical and paramedical acts, will be valued using the tariffs reimbursed by the French health insurance. Visits and paramedical acts will be valued using the French General Nomenclature of Professional Acts. Medical acts will be valued using the French Common Classification of Medical Acts, except for laboratory tests for which valuation will be based on the Nomenclature of Biological Acts. For all these fees, we will apply the corresponding reimbursement rate and we will subtract, if necessary, the medical deductible that is due by the patient and not reimbursed by the French Social Health Insurance (FSHI).

A cost-utility analysis will be performed. This study will establish a link between costs and medical consequences, expressed in QALY gained, at 6 months between the two care management strategies. The ICUR between SSD/AHL patients treated by CI and those that are only followed-up, will be calculated as follows (Drummond et al., 2005):

$$ ICUR=\frac{\Delta  C}{\Delta  U}=\frac{Mean\ cost\ \left(\  CI\right)- Mean\ cost\ (abstention)}{Mean\ QALY\ (CI)- Mean\ QALY\ (abstention)} $$

Where ΔC and ΔU were increments of costs and utilities, respectively.

A Budget Impact Analysis (BIA) will be implemented to measure the net costs to the social health insurance of the care management of SSD/AHL patients, taking into account all positive and negative variations, the use of health resources that could result from the CI [41].

The BIA, which will be conducted using international and French guidelines, will include the predictable variation of context elements (e.g. demographic and epidemiological changes) [41, 42]. The social health insurance perspective will be taken and the time horizon will be 3 years.

The analysis will include three phases:

- An inventory of the care management of SSD/AHL patients,

- A care management model of these patients after the introduction of the new strategy (i.e. CI) to their care,

- The estimated cost for health insurance for these situations and the difference between the two strategies, to estimate the financial impact of the introduction of the new strategy in the treatment of SSD/AHL patients.

The introduction of the strategy based on the CI can change their own cost, but also have an impact on the size of the population (target and reached population), patterns of care performed prior to and after, the unit costs of resources mobilised in the context of strategies. The BIA should take these factors into account.

### Secondary outcome measures

All participants are asked to complete several quality of life questionnaires and hearing tests at inclusion and 6 months after their choice of the treatment (abstention, CROS hearing aids, BAHA, cochlear implantation). Pre/post treatment analyses will be carried out in each of these groups. Comparisons will be made in the group of subjects who chose a CI, randomised between immediate CI and initial observation.

### Quality of life

As described above, a generic evaluation of quality of life is performed using the EuroQol-5D-3 L scale. Hearing-specific quality of life questionnaires will also be administered.

The Nijmegen Cochlear implant questionnaire (NCIQ) contains 60 questions exploring 6 areas concerned with the quality of auditory perception (basic perception, complex perception, speech production, self-esteem, social activities and interactions) [43]. Indeed, the perception of basic environmental sounds such as bells or footsteps is assessed as well as oral communication with new contacts. Each item is formulated as a statement with a 5 point response scale to indicate the degree to which this statement is judged true (from “never”:1 to “always”:5). An extra point can be chosen if none of the points fits.

An evaluation of the discomfort related to the possible tinnitus associated with the deafness will rely on a visual analogue scale (VAS) ranging from 0 to 10. This scale is presented as a 17 cm plastic ruler with a vertical arrow on one side and a graduated scale on the other side (0 to 10 cm). The subject first indicates the level of annoyance generated by the tinnitus on the vertical arrow using a cursor and the corresponding numeric value is reported by the evaluator. Then, the intensity of tinnitus is assessed using another ruler with the same dimensions.

### Speech understanding in noise

Speech recognition in competing noise is measured using the French Matrix test [44] in sound field (IAC 120A-1 sound booth). The French Matrix test is a closed-set sentence test that uses 50 well-known words in French. Each sentence has the same syntactic structure: name - verb - number - object – colour, for example: “Felix draws six blue bikes”. The number of combinations of five words is large enough to eliminate memory-based responses. Speech and noise signals are generated from an IBM PC running the OMA software (www.hoertech.de) and presented via loudspeakers and amplifier (Studio Lab, SLB sat 200). Speech signal is presented at a fixed level of 65 dB SPL and the level of competing noise is adjusted using the adaptive procedure described by Jansen et al. to obtain the signal-to-noise ratio in dB for 50% correct word recognition (SNR50).

SNR50 is obtained in three different spatial configurations: one with speech and noise presented from a single loudspeaker in front of the subject at 0° and two conditions with speech and noise presented from separate loudspeakers at 60° to the left and right of the subject. The choice of a spatial configuration (− 60°, 0° and + 60°) was selected based on previous results [45] showing that when such positions are used, the head-shadow effect is reduced while the binaural unmasking is maximised compared to a ± 90° configuration. The “dichotic” condition is defined as speech presented to the poorer hearing ear and the noise to the contralateral, normal hearing ear; the diotic condition as both the signal and the noise presented from the loudspeaker located in front of the subject; and the reverse dichotic condition with speech presented to the normal hearing ear and noise to the poorer ear. Subjects will be asked to repeat any word which is heard.

### Auditory horizontal localisation

Horizontal localisation ability is assessed using an array of 7 horizontal loudspeakers and an amplifier (Studio Lab, SLB sat 200) located at intervals of 30 degrees from – 90 degrees to 90 degrees in a frontal semicircle diameter of 1.2 m at the subject’s head level. The stimulus comprises two 150 msec white gaussian noises from 20 Hz to 20 kHz with a 0.05 msec ramp. A silence of 150 msec is inserted between the two noises for total stimulus duration of 450 msec. This stimulus is similar to the one used by Slattery and Middlebrooks [46] and is presented 63 times (9 presentations per loudspeaker) with a period of 2 s silence between each presentation to allow the subjects to indicate orally the loudspeaker location. Subjects are asked to not move their head during the experiment: this was controlled by the examiner. Localisation ability measurements are the rate of correct localisation and the root mean square error.

### Statistical methods, data reporting and analysis

Once the validity of the database is double-checked, descriptive analyses will be carried out for each treatment option (observation, CROS hearing aids, bone conduction device, cochlear implant). Quantitative variables will be described as means, standard deviations, and percentile distribution. The number and proportion of subjects will be used to describe categorical data.

Regarding the randomised controlled trial phase comparing CI versus observation, the analysis will be conducted on all subjects who were randomised on an intention-to-treat basis. For each outcome (quality of life, hearing performances indicators), the value measured at 6 months will be compared between the two groups using bivariate analyses (Student’s T test or Wilcoxon rank sum tests) and linear regressions adjusted for the baseline value.

For each intervention, we will assess the associations between changes in hearing performances and changes in quality of life. Data will be presented according to the Consolidated Standards of Reporting Trials (CONSORT) Statement [47, 48].

In order to test the robustness of the ICUR, deterministic and probabilistic sensitivity analyses will be conducted.

As part of the sensitivity analysis, we will determine the robustness of the results by testing the impact on the outcome of the variation of different cost and utility parameters [40]. Probabilistic sensitivity analysis, performed using the non-parametric bootstrap method, identified the uncertainty around the ICUR by estimating its confidence interval [49]. Moreover, a Cost-Utility Acceptability Curve (CUAC) will be built to summarise the impact of uncertainty on the ICUR in relation to possible values of the cost-effectiveness threshold [50].

## Discussion

The detrimental consequences of SSD and AHL have been demonstrated for auditory performances as well as for the quality of life experienced by the patients [8, 51]. However, most studies emphasise the large variability of these consequences, with SSD subjects achieving near-normal localisation accuracy [52–54] or speech understanding in noise [52]. Likewise, the impact on quality of life varies considerably both on specific and generic questionnaires [8, 53] and the role of associated incapacitating symptoms such as tinnitus should be underlined [8].

The present protocol attempts to give account for this heterogeneity through the wide inclusion criteria which have been chosen. The choice of treatment and the outcomes of different subgroups (subjects with incapacitating tinnitus, SSD subjects, AHL subjects, and subjects with long lasting profound deafness) will therefore be specifically described. The theoretical advantage of cochlear implantation over CROS hearing aids and BC implants is to restore an auditory stimulation of the poor ear. This represents the mandatory condition to restore, at least partially, the perception of interaural differences. Further, alleviation of tinnitus is an established side-effect of CI, and subjects with incapacitating tinnitus associated with SSD/AHL might benefit more from this treatment than other SSD/AHL subpopulations.

The current study falls within the framework of validation of SSD/AHL as a new indication of cochlear implantation in France. In this line, the NCIQ was chosen as specific subjective evaluation of hearing before and after treatment, to refer to previous studies on benefits of CI in bilateral severe-to-profound deafness [32, 55–58]. Even though most reports on CI in SSD/AHL used the Speech, Spatial and Qualities of hearing to assess binaural benefits of CI in this population, the NCIQ allows a global evaluation of hearing handicap with several items referring to social limitations and psychological consequences of deafness. Further, this instrument was also used in several studies on CI in SSD/AHL [59].

The calculation of an intervention utility relies on a generic evaluation of quality of life. Among the different instruments which may be used, the Health Utility Index Mark 3 has been identified as sensitive to detect hearing changes after a treatment [51, 60]. Nevertheless, the EQ-5D is still the instrument which is recommended by the French Health Authority (HAS, Haute Autorité de Santé) as well as the preferred instrument recommended by NICE in the UK and the FDA in the USA in the framework of the Health Technology Assessment [61, 62]. It yields an exhaustive measure combining into a single score, both the symptoms of the disease, with its various physical, sensory, cognitive effects and the adverse effects of treatment.

The present study is the first randomised controlled trial on CI in SSD/AHL with the determination of the cost-utility ratio as a primary endpoint. It also provides an assessment of hearing modifications which may be expected from most of treatments for SSD/AHL, such as CROS systems and BAHA. Eventually, it will help delineating the national new criteria for CI indication.

### Trial status

Inclusions completed. Data controls ongoing.

## Abbreviations

AHL: Asymmetric Hearing Loss
BAHA: Bone-Anchored Hearing Aid
BC: Bone conduction
BIA: Budget Impact Analysis
CI: Cochlear Implant
CONSORT: Consolidated Standards Of Reporting Trials
CROS: Controlateral Routing of the Signal
CUAC: Cost-Utility Acceptability Curve
DRG: Disease Related Groups
FDA: Food and Drug Administration
FSHI: French Social Health Insurance
HUI-3: Health Utilities-3
ICUR: Incre mental Cost-utility Ratio
NCIQ: Nijmegen Cochlear Implant Questionnaire
NICE: National Institute for Health and Care Excellence
PTA: Pure Tone Average
SNR: Signal/Noise Ratio
SSD: Single Sided Deafness
SSQ: Speech, Spatial and Qualities Hearing scale
VAS: Visual Analogue Scale
